# Feasibility and Effect of Physiological-Based CPAP in Preterm Infants at Birth

**DOI:** 10.3389/fped.2021.777614

**Published:** 2021-12-03

**Authors:** Tessa Martherus, Kristel L. A. M. Kuypers, Stefan Böhringer, Janneke Dekker, Ruben S. G. M. Witlox, Stuart B. Hooper, Arjan B. te Pas

**Affiliations:** ^1^Division of Neonatology, Department of Pediatrics, Willem-Alexander Children's Hospital, Leiden University Medical Center, Leiden, Netherlands; ^2^Department of Biomedical Data Sciences, Leiden University Medical Center, Leiden, Netherlands; ^3^The Ritchie Centre, Hudson Institute of Medical Research, Melbourne, VIC, Australia; ^4^Department of Obstetrics and Gynecology, Monash University, Melbourne, VIC, Australia

**Keywords:** CPAP, respiratory support, physiology, birth, preterm

## Abstract

**Background:** Preterm infants are commonly supported with 5–8 cmH_2_O CPAP. However, animal studies demonstrate that high initial CPAP levels (12–15 cmH_2_O) which are then reduced (termed physiological based (PB)-CPAP), improve lung aeration without adversely affecting cardiovascular function. We investigated the feasibility of PB-CPAP and the effect in preterm infants at birth.

**Methods:** Preterm infants (24–30 weeks gestation) were randomized to PB-CPAP or 5–8 cmH_2_O CPAP for the first 10 min after birth. PB-CPAP consisted of 15 cmH_2_O CPAP that was decreased when infants were stabilized (heart rate ≥100 bpm, SpO_2_ ≥85%, FiO_2_ ≤ 0.4, spontaneous breathing) to 8 cmH_2_O with steps of ~2/3 cmH_2_O/min. Primary outcomes were feasibility and SpO_2_ in the first 5 min after birth. Secondary outcomes included physiological and breathing parameters and short-term neonatal outcomes. Planned enrollment was 42 infants.

**Results:** The trial was stopped after enrolling 31 infants due to a low inclusion rate and recent changes in the local resuscitation guideline that conflict with the study protocol. Measurements were available for analysis in 28 infants (PB-CPAP *n* = 8, 5–8 cmH_2_O *n* = 20). Protocol deviations in the PB-CPAP group included one infant receiving 3 inflations with 15 cmH_2_O PEEP and two infants in which CPAP levels were decreased faster than described in the study protocol. In the 5–8 cmH_2_O CPAP group, three infants received 4, 10, and 12 cmH_2_O CPAP. During evaluations, caregivers indicated that the current PB-CPAP protocol was difficult to execute. The SpO_2_ in the first 5 min after birth was not different [61 (49–70) vs. 64 (47–74), *p* = 0.973]. However, infants receiving PB-CPAP achieved higher heart rates [121 (111–130) vs. 97 (82–119) bpm, *p* = 0.016] and duration of mask ventilation was shorter [0:42 (0:34–2:22) vs. 2:58 (1:36–6:03) min, *p* = 0.020]. Infants in the PB-CPAP group required 6:36 (5:49-11:03) min to stabilize, compared to 9:57 (6:58–15:06) min in the 5–8 cmH2O CPAP group (*p* = 0.256). There were no differences in short-term outcomes.

**Conclusion:** Stabilization of preterm infants with PB-CPAP is feasible but tailoring CPAP appeared challenging. PB-CPAP did not lead to higher SpO_2_ but increased heart rate and shortened the duration of mask ventilation, which may reflect faster lung aeration.

## Introduction

Historically, elective intubation and mechanical ventilation were standard care in the delivery room (DR), but now respiratory support is primarily given non-invasively to minimize risk of injury (1–3). The effectiveness of non-invasive support is dependent on infants having a patent airway since the larynx of newborn infants closes during apnea (4–8). As the larynx only opens during a breath, support at birth now focuses on stimulating and supporting spontaneous breathing (9). Recent studies showed that breathing effort can be stimulated by adequate oxygenation, repetitive tactile stimulation and caffeine (10–12). However, ongoing breathing activity is totally reliant on lung aeration and these interventions do not necessarily enhance lung aeration (13). Respiratory support in the DR can further be optimized by improving lung aeration.

Lung aeration is driven by the transpulmonary pressure, which is a pressure gradient generated during inspiration (14–16) and can be increased by applying continuous positive airway pressure (CPAP) to the mouth opening. Preterm infants are routinely supported with 5–8 cmH_2_O CPAP in the DR, yet this strategy has been extrapolated from care used in the neonatal intensive care unit (NICU). However, in the NICU, CPAP is used to support infants hours to days after birth when the lungs are well-aerated (17, 18). There is little evidence that a CPAP of 5–8 cmH_2_O is the optimal pressure range to promote lung aeration when infants have a liquid-filled highly incompliant lung at birth.

**Figure 1:**
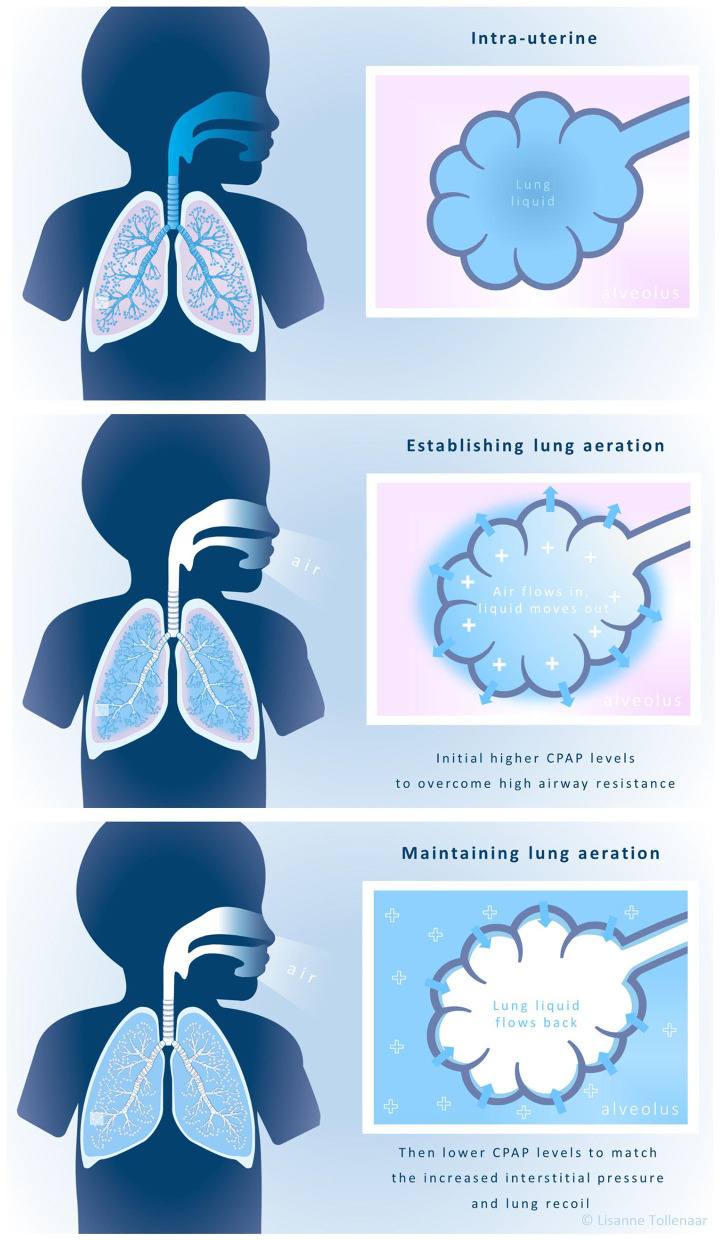
Physiological-based CPAP.

Physiological based (PB)-CPAP takes the changes that are required to transition from fetus to newborn infant into consideration (Figure 1). When infants are born, their airways are filled with liquid that needs to be replaced with air. Initially, the role of CPAP is to assist liquid movement from the airways into the interstitial tissue during inspiration, by increasing the pressure gradient across the airway wall (19, 20). High pressures are needed to overcome the high airway resistance generated by the viscosity of liquid and its movement across the epithelium (14–16). Once lung aeration is established, the role of CPAP changes to maintaining lung aeration. Lower CPAP levels are then likely sufficient to prevent liquid re-entry and alveolar collapse at end-expiration (14–16, 19–24). As real time parameters guide how caregivers decrease CPAP levels, PB-CPAP is tailored to each individual and CPAP levels will suit the different phases of the neonatal transition.

PB-CPAP has so far only been investigated in a preclinical setting. Preterm rabbit (25) and sheep (26) studies demonstrated that initially, 15 cmH_2_O CPAP improves lung aeration, facilitates cardiovascular stability and better supports spontaneous breathing compared to the currently used CPAP levels. Lung aeration and breathing rates could be maintained if CPAP was gradually decreased to at least 8 cmH_2_O but did increase oxygen requirements. Overall, there were no indications that PB-CPAP impedes the cardiovascular system or increases the risk on pneumothorax, CPAP belly or intraventricular hemorrhages.

This is the first clinical study that investigates PB-CPAP in preterm infants and we aimed to test the feasibility of using this strategy in the DR and evaluate the effect on physiological parameters.

## Methods

This single-blinded randomized controlled trial was conducted at the Leiden University Medical Center (LUMC). Preterm infants born between 24^+0^ and 29^+6^ weeks gestation were eligible for inclusion. Exclusion criteria were congenital malformations or abnormalities (observed during pregnancy) that affect the transition at birth. Parents were not approached for study participation if there was a language barrier, if there was no time to acquire informed consent or if it was considered inappropriate.

Infants were randomized to PB-CPAP or 5–8 cmH_2_O CPAP prior to the delivery using an electronic data capture system (Castor EDC, Amsterdam, the Netherlands). Twin pregnancies were randomized as pairs. While allocation was initially solely stratified by gestational age [24^+0^-26^+6^ and 27^+0^-29^+6^ weeks, variable block sizes (4–6)], the stratification number of infants per pregnancy (single and twin pregnancies) was added after randomizing twenty-three infants in November 2020 and documented in a protocol amendment.

Infants randomized to PB-CPAP received 15 cmH_2_O CPAP until they reached all predefined stabilization criteria (heart rate ≥100 bpm, SpO_2_ ≥85%, FiO_2_ ≤ 0.4, spontaneous breathing), then CPAP was decreased to 8 cmH_2_O in three steps (2–2-3 cmH_2_O/min). The decrease in CPAP only continued if the infant still met the stabilization criteria. If infants became apneic, intermittent positive pressure ventilation (iPPV) was initiated with a PEEP of 8 cmH_2_O. Once infants continued on CPAP after a period of iPPV, the pressure was increased back to the CPAP level that had been used prior to the ventilation period. After completing the 10 min study duration, all infants continued with CPAP levels conforming to local protocols. Infants randomized to PB-CPAP could also receive an escape strategy if deemed appropriate by the caregiver. We assumed that infants who were breathing sufficiently prior to the start of respiratory support, would have already established lung aeration. Therefore, continuous 8 cmH_2_O CPAP would be sufficient to maintain aeration. Infants supported with this escape strategy were still included in the PB-CPAP group during the analysis. Infants randomized to the control group, received 5–8 cmH_2_O CPAP. Remaining procedures were executed in line with the local protocol, with exception of infants who participated in the ABC3 study (NCT0380851) and were randomized to physiological based cord clamping. In our local resuscitation guideline, iPPV is initially given non-invasive via mask. However, caregivers can change the interface and apply iPPV via endotracheal intubation if required. Furthermore, the decision to administer caffeine remains at the discretion of the caregiver.

To record SpO_2_ and heart rate, a Radical-7 Masimo SET pulse oximeter probe (Masimo Corporation, CA, USA) was placed around the infant's right wrist. The Teledyne Oxygen Analyser AX300-I (Teledyne Analytical Instruments, CA, USA) inserted into the inspiratory limb of the Neopuff™ circuit measured fraction of inspired oxygen (FiO_2_), while the disposable Avea Varflex Flow transducer (Carefusion, CA, USA) connected between the Neopuff™ and the facemask measured flows and pressures. Signals were collected by the New Life BOX Neo-RDS (Applied Biosignals, Weener, Germany) and saved by Polybench software (Applied Biosignals, Weener, Germany). Pulmochart software (Applied Biosignals, Weener, Germany) allowed a breath-by-breath analysis to calculate breathing parameters, corrected for birth weight.

The primary outcomes were feasibility and SpO_2_ in the first 5 min after birth. Feasibility was explored by evaluating the data from the New Life Box Neo-RDS and videos of resuscitations focusing on protocol adherence and via post-trial evaluations with neonatologists. The neonatologists evaluated this trial individually, as they were asked about their general understanding and experience with this study and obstacles and possible improvements with regard to the PB-CPAP protocol specifically. Physiological outcomes included SpO_2_, FiO_2_, SpO_2_/FiO_2_ ratio, heart rate, duration of hypoxia [SpO_2_ <25th percentile of Dawson's target ranges (27)] and bradycardia (heart rate <100 bpm) during the first 5 and 10 min after birth. Respiratory effort parameters included breathing rate, inter-breath interval variability, minute volume, inspired tidal volume, peak inspiratory flow rate (PIFR) and the use of iPPV and caffeine. The infant's overall stability was reflected by Apgar scores and time until stabilization (defined as above). Short-term outcomes included intubation in the DR, intubation <24 h, pneumothorax <5 days, pulmonary hemorrhage, surfactant administration, intraventricular hemorrhages (IVH), spontaneous intestinal perforations and death before NICU discharge. Collected demographical characteristics were gestational age, birth weight, gender, type of pregnancy, mode of delivery, time of cord clamping, 1 min Apgar score, antenatal corticosteroids (full course defined as two doses administered at least 24 h, but at a maximum of 2 weeks prior to delivery), complications during pregnancy and maternal medication use.

The sample size calculation is based on infants who were born in the LUMC, participated in DR studies (11, 28) and received 5–8 cmH_2_O CPAP. Infants (*n* = 78) achieved a mean SpO_2_ of 59% ± 13 in the first 5 min after birth. An increase to 72% was considered to be clinically relevant and for this a sample size of 32 infants would be required [α = 0.05, power (1–β) = 0.8, 2-sided]. Because we randomized per pregnancy and our study population is enriched with twin pregnancies (with the LUMC being the national referral center for complicated twin pregnancies), an additional number of infants needed to be included to prevent loss of power. In February 2019, 28.5% infants included in the MONitoR trial (29) were twins and showed an intra-class correlation of 0.586 for SpO_2_. Therefore, our sample size required an additional 16.7% (0.285^*^0.586^*^100). Anticipating 10% drop-outs due to technical errors or study withdraw, a sample size of 21 infants per group was anticipated.

Statistical analysis was performed using SPSS software version 25.0 (IBM, Chicago, Illinois, 2021). Outcomes were analyzed per group considering the number of included infants, despite stratification criteria. Data were presented as median (IQR) or number (%). When continuous data covered a time period, a mean was calculated for that specific time period per individual. The individual means were then used to calculate and present the group median (IQR). *P*-values < 0.05 were considered statistically significant.

The primary outcome SpO_2_ was compared over time using a linear mixed-effect regression model, accounting for the relation between multiple measurements of the same infants. Physiological effects of the CPAP strategies were examined in a per-protocol analysis that excluded infants who were randomized to PB-CPAP but rather received 5–8 cmH_2_O CPAP. Additional intention-to-treat and a sensitivity analysis were performed to test the robustness of the study outcomes. Fixed effects in the regression models were group, time and the interaction group^*^time. *P*-values of the group variable were used to determine the results of the primary outcome while graphical representations have been used to illustrate the effects of randomization groups over time.

Demographic and secondary outcomes were analyzed as per-protocol. Area under the curve (AUC) was calculated for outcomes over time wherein missing values were replaced by the mean, previous or following measurement. Numerical outcomes were analyzed using a Mann-Whitney *U* test, whereas categorical outcomes were analyzed using a Fisher's exact test or a Logrank test.

## Results

**Figure 2:**
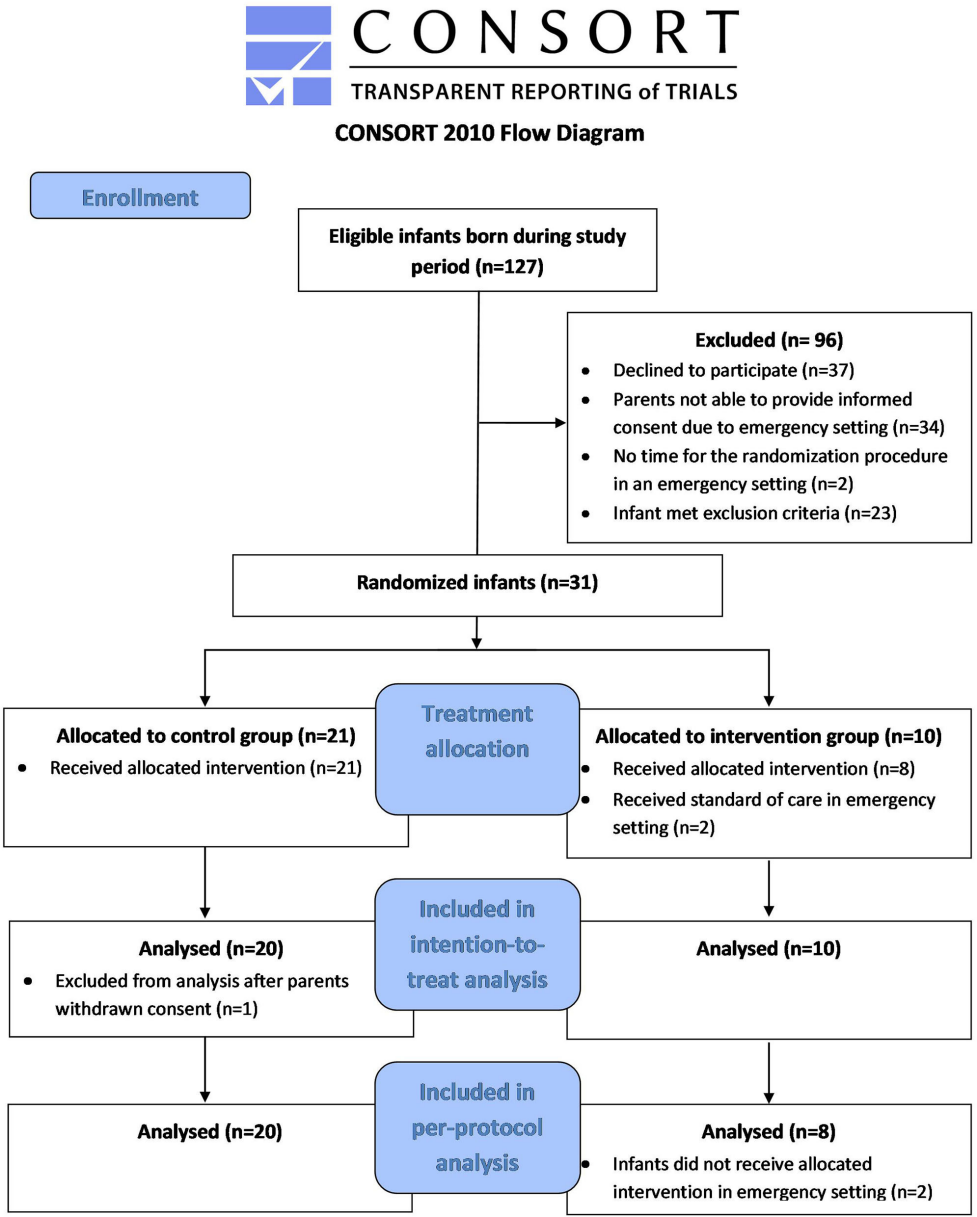
Consort 2010 flowchart.

One hundred and twenty-seven eligible infants were born in the LUMC during the study enrolment period from October 2019 until March 2021, with the study being halted from March 2020 until May 2020 due to COVID-19 restrictions. Ninety-six infants were not included as they met exclusion criteria (*n* = 23), parents declined to participate (*n* = 37), there was insufficient time to ask for study participation (*n* = 34) or there was insufficient time to complete the randomization procedure (*n* = 2) in an emergency setting. Thirty-one infants were randomized. One infant was excluded from the analysis due to withdrawal of parental consent, leaving thirty infants for inclusion in the intention-to-treat analysis (PB-CPAP *n* = 10, 5–8 cmH_2_O *n* = 20). There was only one twin pair randomized to PB-CPAP (by chance). As this twin pair received 5–8 cmH_2_O in an emergency setting, these infants were excluded from the per-protocol analysis due to protocol violation. Therefore, twenty-eight infants were included in the per-protocol analysis (PB-CPAP *n* = 8, 5–8 cmH_2_O *n* = 20, Figure 2). In the PB-CPAP group, seven infants were supported with the initial 15 cmH_2_O CPAP that was gradually decreased to 8 cmH_2_O, while one infant received the escape strategy of continuous 8 cmH_2_O CPAP.

Table 1 shows baseline characteristics for both groups. Infants in the PB-CPAP group had a median (IQR) gestational age of 26^+5^ (25^+4^-27^+4^) weeks, whereas infants in the 5–8 cmH_2_O group were 28^+5^ (25^+4^-29^+4^) gestational age (*p* = 0.601). There were no statistical differences with regard to birth weight, gender, mode of delivery and the use of antenatal steroids. The number of twin pregnancies was significantly higher in the 5–8 cmH_2_O CPAP group (50%) as compared to the PB-CPAP group (0%, *p* = 0.025). There were no significant differences regarding maternal medication use, complications that occurred during the pregnancy or physiological based cord clamping that could have affected the respiratory effort at birth. Apgar scores at 1 min were similar between groups.

**Table 1:** Demographical data (per-protocol).

	**PB-CPAP (*n* = 8)**	**5–8 cmH_**2**_O CPAP (*n* = 20)**	***p*-value**
Gestational age at birth (weeks)^a^	26^+5^ (25^+4^-27^+4^)	28^+5^ (25^+4^-29^+4^)	0.601
Birth weight (grams)^a^	1,022 (835–1,255)	935 (757–1,180)	0.409
Gender (% male)^b^	5 (63%)	12 (60%)	1.000
Type of pregnancy (*n*, % twin)^b^	0 (0%)	10 (50%)	0.025
Mode of delivery (*n*, % cesarean section)^b^	1 (13%)	10 (50%)	0.099
Antenatal steroids Course started (*n*, %)^b^	8 (100%)	18 (90%)	1.000
Course completed (*n*, %)^b^	6 (75%)	13 (65%)	1.000
Maternal medication use influencing infants respiration e.g., general anesthesia (*n*, %)^b^	0 (0%)	1 (5%)	1.000
Complications during pregnancy (*n*, %)^b^	3 (38%)	11 (55%)	0.678
Preterm pre-labour rupture of membranes (*n*, %)	2 (25%)	6 (30%)	
Pregnancy-induced hypertension (*n*, %)	1 (13%)	4 (20%)	
Intra-uterine infection (*n*, %)	1 (13%)	5 (25%)	
Intra-uterine growth restriction (*n*, %)	0 (0%)	3 (15%)	
Multiple (*n*, %)	1 (13%)	7 (35%)	
Physiological based cord clamping (*n*, %)^b^	3 (38%)	5 (25%)	0.651
Apgar score ‘1^a^	6 (2–7)	6 (3–8)	0.823

a *Demographical data analyzed as per-protocol. Numerical data presented as median (Q1–Q3) compared using a Kruskal-Wallis test*.

b *Categorical data presented as n, (%) compared using a Fisher's exact test*.

### Feasibility of the Current PB-CPAP Strategy

#### Protocol Adherence

Protocol adherence could not be evaluated in all infants due to technical errors. Protocol adherence was evaluated in 7/8 infants of the PB-CPAP group and three minor protocol deviations were found. One infant received three inflations with a PEEP of 15 cmH_2_O and in two infants CPAP was decreased faster than described in the study protocol. When protocol adherence was evaluated in 18/20 infants of the 5–8 cmH_2_O group, it was found that three infants received a CPAP/PEEP level of 4, 10, and 12 cmH_2_O unintentionally for several minutes.

#### Post-trial Evaluations

Evaluations showed that although all caregivers supported the concept of PB-CPAP, only a few (3/11) felt comfortable in performing the protocol. While caregivers often use CPAP, monitor parameters and adjust settings (e.g., FiO_2_), the PB-CPAP protocol was considered too complex using existing equipment due to the many predefined actions and evaluation moments. If infants became apneic, CPAP was decreased from 15 to 8 cmH_2_O during iPPV, increased back to 15 cmH_2_O once CPAP was continued and was decreased step-wise to 8 cmH_2_O once infants were stabilized. Caregivers indicated that it was challenging to perform these CPAP changes while providing stabilization and a dedicated person (who focused on CPAP) was required to ensure protocol adherence.

### Effects of PB-CPAP

#### Effect on Physiological Parameters

**Figure 3:**
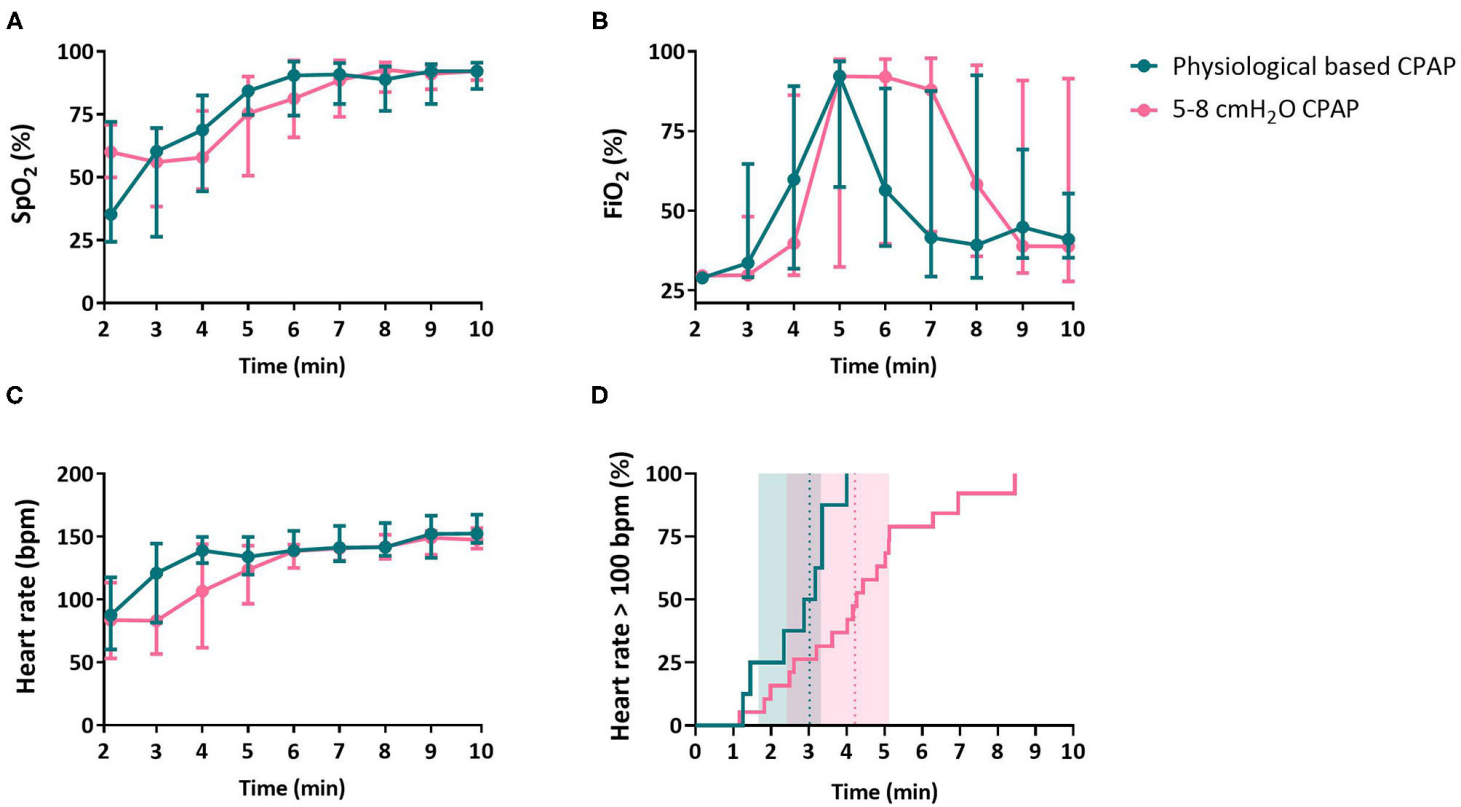
Physiological outcomes. Physiological outcomes oxygen saturation [SpO_2_, **(A)**], fraction of inspired oxygen [FiO_2_, **(B)**], heart rate **(C)** and time that heart rate exceeds 100 bpm **(D)** of infants receiving PB-CPAP and 5–8 cmH_2_O CPAP following the per-protocol analysis. In **(D)**, heart rate >100 bpm is illustrated as the incidence over time (continuous line) with the median group time (dotted vertical line) and (shaded) inter quartile range.

The SpO_2_ in the first 5 min after birth was not significantly different between groups in the per-protocol [PB-CPAP vs. 5–8 cmH_2_O CPAP, 61 (49–70) vs. 64 (47–74)%, variance of random intercept 128.7, variance of residual 307.3, *p* = 0.973, Figure 3A] and the intention-to-treat analysis [62 (52–70) vs. 64 (47–74)%, variance of random intercept 123.3, variance of residual 305.9, *p* = 0.992, Supplementary Table]. There were no significant differences between groups in SpO_2_ (Figure 3A), FiO_2_ (Figure 3B) and the SpO_2_/FiO_2_ ratio. However, infants supported with PB-CPAP achieved significantly higher heart rates in the first 5 min [121 (111–130) vs. 97 (82–119) bpm, *p* = 0.016] and tended to have higher heart rates in the first 10 min after birth [135 (127–141) vs. 123 (107–136) bpm, *p* = 0.075] (Figure 3C). Infants stabilized with PB-CPAP required significantly less time to achieve a stable heart rate >100 bpm [03:01 (01:40–03:19) vs. 04:13 (02:25–05:07) min, *p* = 0.009, Figure 3D] (Table 2).

**Table 2:** Parameters of physiology and respiratory effort in the delivery room.

	**PB-CPAP (*n* = 8)**	**5–8 cmH_**2**_O CPAP (*n* = 19)**	***p*-value**
**Physiological parameters**			
SpO_2_ (%)	61 (49–70)	64 (47–74)	0.973
Min 2–5 after birth^a^			
Min 2–10 after birth^b^	76 (67–86)	80 (65–82)	0.815
SpO_2_ > 80% at 5 min after birth (%)^*^^c^	4/7 (57%)	7/17 (42%)	0.659
Duration of hypoxia 5 min after birth (%)^b^	34 (27–58)	34 (22–65)	1.000
Duration of hypoxia 10 min after birth (%)^b^	62 (34–87)	59 (38–82)	1.000
FiO_2_ (%)			
Min 2–5 after birth^b^	55 (37–71)	49 (31–65)	0.585
Min 2–10 after birth^b^	49 (41–73)	65 (36–77)	1.000
SpO_2_/FiO_2_ ratio			
Min 2–5 after birth^b^	1.08 (0.79–1.18)	1.50 (0.94–2.43)	0.549
Min 2–10 after birth^b^	1.79 (1.19–1.96)	1.37 (0.94–2.50)	0.798
Heart rate (bpm)	121 (111–130)	97 (82–119)	0.016
Min 2–5 after birth^b^			
Min 2–10 after birth^b^	135 (127–141)	123 (107–136)	0.075
Heart rate exceeds 100 bpm (min:s)^d^	03:01 (01:40–03:19)	04:13 (02:25–05:07)	0.005
**Parameters of respiratory effort**			
Breathing rate (breaths/min)			
Min 2–5 after birth^b^	29 (17–39)	23 (17–29)	0.389
Min 2–10 after birth^b^	37 (20–42)	28 (24–33)	0.458
Inspiratory tidal volume (mL/kg)			
Min 2–5 after birth^b^	2.2 (2.0–2.7)	2.4 (0.1–4.9)	0.836
Min 2–10 after birth^b^	2.6 (2.4–4.0)	2.9 (0.6–6.0)	0.929
Minute volume (mL/kg/min)			
Min 2–5 after birth^b^	84 (64–170)	105 (5–201)	0.929
Min 2–10 after birth^b^	120 (62–187)	114 (24–212)	1.000
PIFR min 4–10 after birth (L/kg/min)^b^	0.94 (0.60–1.38)	0.92 (0.37–2.01)	0.836
Inter–breath interval variability min 2–10 after birth (%)^b^	117 (86–169)	76 (55–138)	0.357
Intermittent positive pressure ventilation			
Incidence (%)^c^	6 (75%)	11 (55%)	0.419
Start (min:s)^b^	3:00 (2:08–4:46)	2:27 (2:04–3:16)	0.462
Duration (min:s)^b^	0:42 (0:34–2:22)	2:58 (1:36–6:03)	0.020
Caffeine Incidence (%)^c^	2 (25%)	11 (55%)	0.221
Time of administration (min:s)^b^	10:26 (08:59–10:26)	11:55 (6:36–15:00)	0.641
**Infant's overall stability**			
Apgar score ‘5^b^	8 (6–8)	8 (6–9)	0.438
Apgar score'10^b^	9 (8–9)	9 (8–9)	0.746
Time until stabilization from birth (min:s)^b^	6:36 (5:49–11:03)	9:57 (6:58–15:06)	0.256

a *Numerical data presented as median (Q1–Q3) compared using a linear regression mixed model or*

b *Kruskal-Wallis test*.

c *SpO_2_ in the first 5 min after birth calculated had a variance of random intercept 128.7, variance of residual 307.3. Categorical data presented as n, (%) compared using a c Fisher's exact test or*

d *Log-Rank survival test*.

*Duration is expressed as % instead of minutes as it considered the % of time that data was available. PIFR is calculated in min 4–10 because of data availability*.

* *Some patients had missing SpO_2_ values at 5 min after birth. Continuous Positive Airway Pressure (CPAP), Expired tidal volume (Vt_e_), Fraction of Inspired Oxygen (FiO_2_), Oxygen saturation (SpO_2_), Peak Inspiratory Flow Rate (PIRF). Hypoxia was measured as SpO_2_ <25^th^ percentile of Dawson target range*.

#### Effect on Respiratory Effort

**Figure 4:**
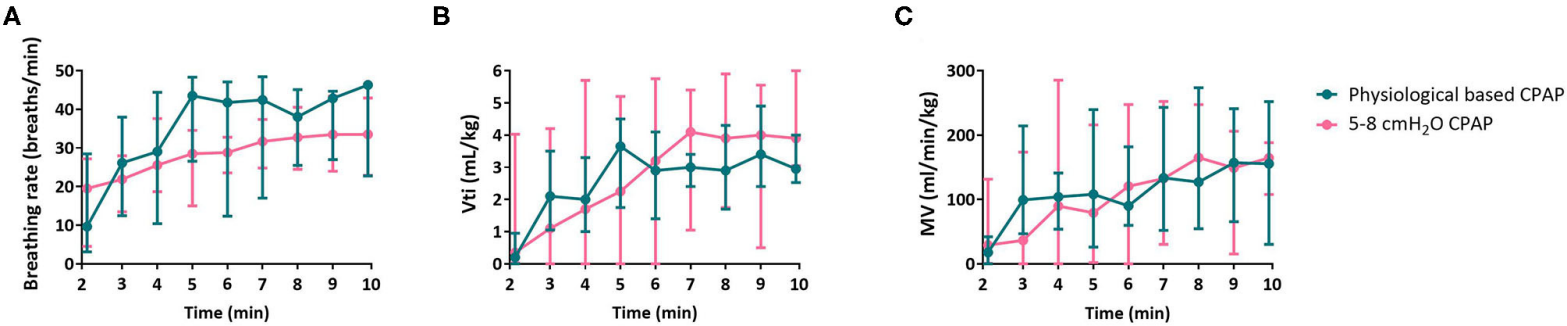
Outcomes of respiratory effort. Outcomes of respiratory effort breathing rate **(A)**, inspiratory tidal volume [Vti, **(B)**] and minute volume [MV, **(C)**] of infants receiving PB-CPAP and 5-8 cmH_2_O CPAP following the per-protocol analysis.

The groups showed no significant differences regarding breathing rate [37 (20–42) vs. 28 (24–33) breaths/min, *p* = 0.458, Figure 4A], tidal volume [2.6 (2.4–4.0) vs. 2.9 (0.6–6.0) mL/kg, *p* = 0.929, Figure 4B] and minute volume [120 (62–187) vs. 114 (24–212) mL/kg/min, *p* = 1.000, Figure 4C]. There were no differences in inter-breath interval variability or peak inspiratory flow rate (Table 2).

There were no significant differences in the number of infants receiving caffeine (25 vs. 55%, *p* = 0.221) or iPPV (75 vs. 55%, *p* = 0.419), yet the duration of mask ventilation was significantly shorter in those supported with PB-CPAP [0:42 (0:34–2:22) min] as compared to 5–8 cmH_2_O CPAP [2:58 (1:36–6:03) min, *p* = 0.020]. In the PB-CPAP group, two infants started to breathe spontaneously during mask ventilation and continued afterwards until CPAP was increased back to 15 cmH_2_O. These infants then stopped breathing and required interventions to re-start spontaneous breathing (Table 2).

#### Outcomes Reflecting the Infant's Overall Stability

The groups showed similar Apgar scores at 5 and 10 min after birth. Infants were considered stable after 6:36 (5:49–11:03) min in the PB-CPAP group and 9:57 (6:58–15:06) min in the 5–8 cmH_2_O CPAP group (*p* = 0.256) (Table 2).

#### Mean Airway Pressure and Mask Leak

**Figure 5:**
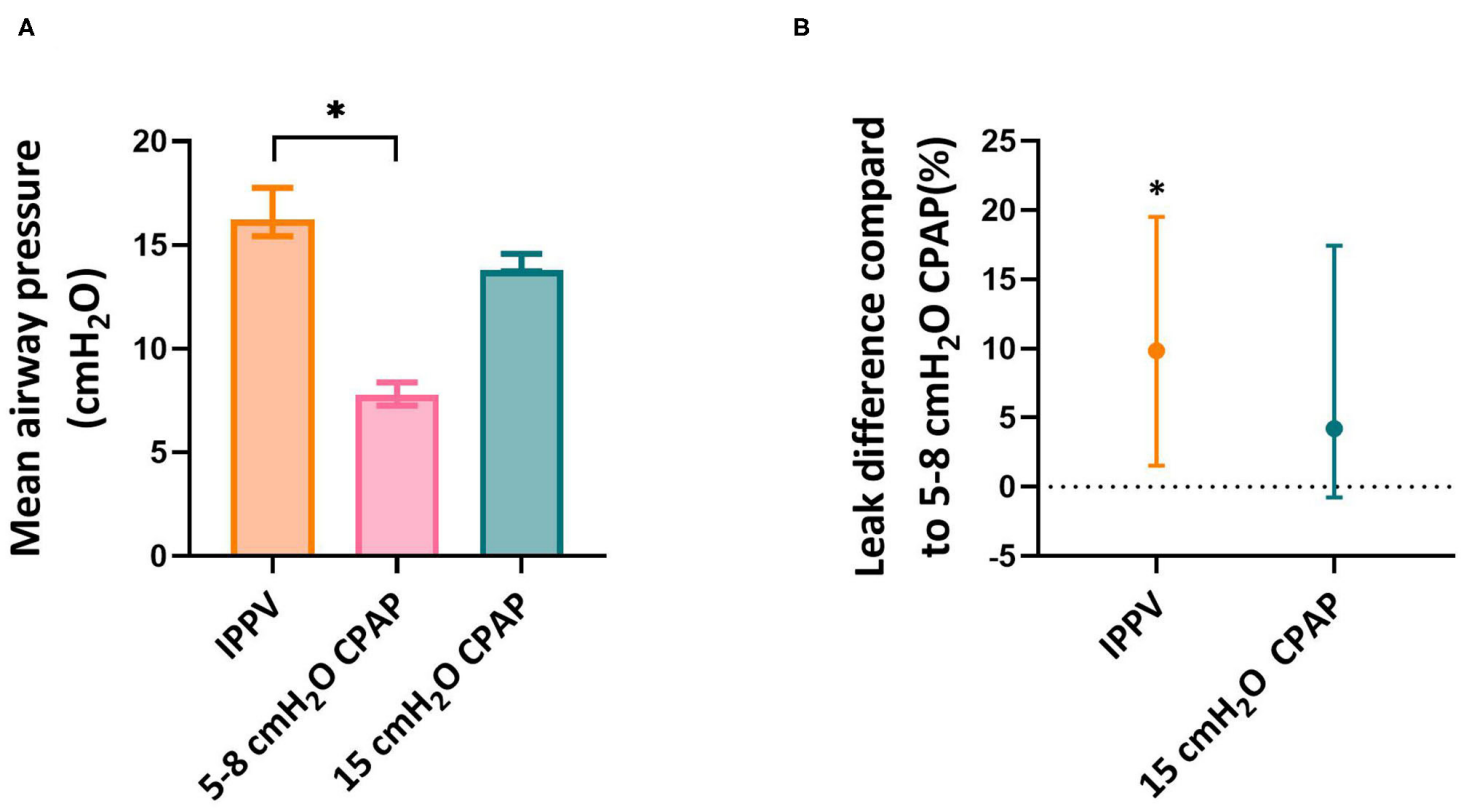
Mean airway pressure and leak. Mean airway pressure **(A)** and leak **(B)** per respiratory support mode. Leak per respiratory support mode is calculated as difference in leak compared to 5–8 cmH_2_O CPAP as it is calculated within infants. Asterisk indicates significant difference.

Comparing the modes of respiratory support, mean airway pressures (MAP) were significantly higher during iPPV [16.2 (15.4–17.8) cmH_2_O] than during 5–8 cmH_2_O CPAP [7.8 (7.3–8.4) cmH_2_O] but did not differ with 15 cmH_2_O CPAP [13.8 (13.7–14.6) cmH_2_O, *p* = 0.006]. During iPPV, there was significantly more mask leak than when 5-8 cmH_2_O CPAP was given with the difference being 10 (2–20)% (*p* = 0.010). There was no significant difference in the amount of leak created during 15 cmH_2_O and 5–8 cmH_2_O CPAP [leak difference, 4 (−1 to 17) %, *p* = 0.345] (Figure 5).

#### Short-Term Clinical Outcomes

There were no differences in short-term neonatal respiratory outcomes including incidences of pneumothorax <5 days, intubation in the DR, intubation <24 h, surfactant administration or pulmonary hemorrhages. Groups showed no statistical significance regarding the incidences of spontaneous intestinal perforations, IVH and/or neonatal mortality (Table 3).

**Table 3:** Short-term clinical outcomes.

	**PB-CPAP (*n* = 8)**	**5–8 cmH_**2**_O CPAP (*n* = 20)**	***p*-value**
Pneumothorax <5 days after birth (%)	0 (0%)	1 (5%)	1.000
Intubation in the delivery room (%)	0 (0%)	2 (10%)	1.000
Intubation <24 h after birth (%)	1 (13%)	5 (25%)	0.640
Surfactant administration (%)	2 (25%)	10 (50%)	0.401
Pulmonary hemorrhages (%)	0 (0%)	0 (0%)	1.000
Spontaneous intestinal perforations (%)	0 (0%)	0 (0%)	1.000
Intraventricular hemorrhages All grades (%)	4 (50%)	5 (25%)	0.371
≥grade III (%)	2 (25%)	1 (5%)	0.188
Neonatal mortality (%)	1 (13%)	4 (20%)	1.000
Combined outcome intraventricular hemorrhages ≥ grade III and/or death (%)	2 (25%)	4 (20%)	1.000

*Categorical data presented as n, (%) compared using a Fisher's exact test. When not specified, outcomes are reported at NICU discharge*.

## Discussion

This study was the first to evaluate the feasibility and the direct effect of PB-CPAP for preterm infants in the DR. The study was halted prematurely due to low inclusion rates and recent changes in our local guideline with regard to initial FiO_2_ levels that conflicted with the study protocol. Although the protocol adherence was high, evaluations by caregivers after the trial indicated that the current PB-CPAP approach is feasible in a research setting but requires simplification before it can be used routinely. Although PB-CPAP did not improve oxygenation, it seemed beneficial for preterm infants as they showed increased heart rate and shortened duration of mask ventilation which reflects a faster and/or improved lung aeration.

The feasibility of our current PB-CPAP approach was evaluated by protocol adherence and post-trial evaluations. There were three minor protocol deviations in the PB-CPAP group, despite the presence of a dedicated person present in the DR who focused on CPAP support. Post-trial evaluations showed that the current approach is too complex. Although routine use of PB-CPAP will likely improve dexterity and the sense of competence among caregivers, the approach requires simplification which can be achieved in various manners. First, the escape strategy (consistent 8 cmH_2_O CPAP) seems redundant and could be removed. The condition for using the escape strategy was good breathing effort, however we now know that this is not reflective of lung aeration (30) and infants with good breathing efforts may still benefit from PB-CPAP. Second, the number of predefined evaluation moments could be reduced by leaving the decision to adjust CPAP levels to the discretion of the caregiver. Third, a consistent CPAP level could be used until the infant stabilizes and interfaces are switched and/or the infant is transferred to the NICU. This would be the most pragmatic option and is already common practice in some centers. While details of how PB-CPAP can be used may differ between centers depending on how it best fits into the overall DR care, early involvement of the medical team and scenario trainings may increase the usability of PB-CPAP.

We hypothesized that PB-CPAP would improve lung aeration and subsequently improve physiological parameters but found no effects on SpO_2_ or FiO_2_. Recent rabbit studies have demonstrated that the increase in lung aeration and oxygenation are not necessarily co-dependent (25, 30), yet they are likely to be additive and at least some lung aeration is essential. Aeration must positively affect SpO_2_ but the relative contribution of aeration vs. the gradient for oxygen diffusion is complex and influenced by other factors such as pulmonary blood flow and cardiac output. Previous studies in preterm sheep showed that 8 and 15 cmH_2_O CPAP improved oxygenation and lowered FiO_2_ requirements as compared to 5 cmH_2_O CPAP (26, 31). We found no effect on oxygenation in this study, however the effect of PB-CPAP on SpO_2_ could have been diminished by the large difference in gestational age, high FiO_2_ levels in both groups and the fact that the power requirements with respect to sample size could not be met. The actual effect of CPAP on oxygenation in preterm infants remains inconclusive.

PB-CPAP led to a larger increase in heart rate, which may reflect a better lung aeration. When infants are born and aerate their lungs, this stimulates a very large increase in pulmonary blood flow. Recent evidence suggests that as lung liquid moves into the interstitial tissue it triggers J-receptors located in the alveolar wall (15, 23). Stimulation of these receptors is thought to initiate a vagal reflex facilitating global pulmonary vasodilation and a subsequent increase in pulmonary blood flow and heart rate (32, 33). The outcomes of this study resemble preclinical studies demonstrating that 15 cmH_2_O CPAP improves lung aeration (25), PBF and heart rate (26) compared to the currently used CPAP levels. Improved lung aeration would explain why infants required a shortened duration of mask ventilation. Although the time until stabilization was not significantly different between groups, it was striking that infants were stabilized 3 min earlier in the PB-CPAP group.

Two infants restarted breathing during iPPV but stopped when CPAP was increased to 15 cmH_2_O. We speculate that these infants had already established lung aeration and apnea had been caused by a Hering-Breuer reflex or trigeminal reflex (34). Similar findings have been described in preterm rabbits that had established lung aeration, but became apneic as CPAP was suddenly increased from below to above 7 cmH_2_O (4). While this is speculative, some infants may establish aeration during iPPV (25) and future studies have to investigate if increasing CPAP levels after iPPV may induce apnea in some infants. Preferably, CPAP is guided by lung aeration, this cannot be measured during the stabilization of preterm infants yet.

The small number of infants included in this study prevents us from making appropriate conclusions regarding safety. We can only note that there were no direct signs of harm, as there were no pneumothoraxes in the PB-CPAP group. In preclinical studies (25, 26) there were no indications that PB-CPAP causes adverse events at birth. We hypothesize that PB-CPAP does not cause lung over-expansion because the lungs are liquid-filled during the initial stage of the transition (creating a relatively high airway resistance) and due to the involvement of the larynx during non-invasive support. A recent sheep study (26) demonstrated that the larynx is involved in pressure transmission to the lungs during spontaneous breathing and can protect the lungs from overexpansion. Also, the pressure given during PB-CPAP are comparable to the pressures generated by the current DR respiratory support approaches, as MAPs of ~15 cmH_2_O are a common occurrence when mask ventilation includes intermittent positive pressure ventilation (35). Larger clinical trials are needed to examine if PB-CPAP is indeed harmless for preterm infants at birth.

The main limitation of this study is the number of included infants, which prevents us from making appropriate conclusions. While this was caused by various problems that occurred during the trial, a particular problem was low consent rate as 37 parents declined and 33 parents consented (31 randomized, 2 parents gave consent but there was insufficient time to perform the randomization procedure). We found that it was difficult for parents to comprehend the complexity of the procedure as CPAP was an abstract concept for them. Potentially, this selection might lead to bias but we have no indication of difference between the patient cohorts (consent vs. no consent). Since we did not observe adverse effects in this study, deferred consent could be considered for the next study to increase recruitment and decrease the risk of bias.

## Conclusion

This study demonstrated that PB-CPAP may be beneficial but that our current approach is too complex. We were unable to demonstrate if PB-CPAP improves oxygen saturation. Nevertheless, PB-CPAP did increase heart rate and shortened the duration of iPPV, which is presumably the result of improved lung aeration. Short-term neonatal outcomes were similar between groups, however due to the low number of included infants it is not possible to make appropriate conclusions from our study. Future studies may continue investigating PB-CPAP using a simplified version of the current approach.

## Data Availability Statement

The raw data supporting the conclusions of this article will be made available by the authors, without undue reservation.

## Ethics Statement

The studies involving human participants were reviewed and approved by Medical Research Ethics Committee (METC) Leiden-Den Haag-Delft. Written informed consent to participate in this study was provided by the participants' legal guardian/next of kin.

## Clinical Trial Registration

This study was registered in the Netherlands Trial Register (www.trialregister.nl), titled CPAP titration at birth (NL8089).

## Author Contributions

TM, SB, AtP, and SH made substantial contributions to conception and design of the study. TM and KK set-up the study and provided the data collection. TM and SB were responsible for data analysis. TM, KK, JD, SB, and AtP were involved in the data interpretation. TM, AtP, and SH drafted the first version of the manuscript. All authors provided feedback and approved the final version of the manuscript.

## Funding

AtP is a recipient of a NWO innervational research incentives scheme (VIDI 91716428).

## Conflict of Interest

The authors declare that the research was conducted in the absence of any commercial or financial relationships that could be construed as a potential conflict of interest.

## Publisher's Note

All claims expressed in this article are solely those of the authors and do not necessarily represent those of their affiliated organizations, or those of the publisher, the editors and the reviewers. Any product that may be evaluated in this article, or claim that may be made by its manufacturer, is not guaranteed or endorsed by the publisher.

## Abbreviations

CPAP: Continuous Positive Airway Pressure
FiO_2_: Fraction of inspired Oxygen
iPPV: intermittent Positive Pressure Ventilation
IQR: Inter quartile Range
IVH: Intra Ventricular Hemorrhage
LUMC: Leiden University Medical Center
MAP: Mean Airway Pressure
NICU: Neonatal Intensive Care Unit
PEEP: Positive End-Expiratory Pressure
RFM: Respiratory Function Monitor
SD: Standard Deviation
SpO_2_: Oxygen Saturation.
