# Supplementary material for: Feasibility and Effect of Physiological-Based CPAP in Preterm Infants at Birth
Source: Front Pediatr. 2021 Dec 3;9:777614. doi: 10.3389/fped.2021.777614 (PMC8678466; doi:10.3389/fped.2021.777614)
Supplement: Supplementary file 1 [file Table_1.pdf]

SUPPLEMENTARY

Intention-to-treat analysis

	PB-CPAP (n=10)	5-8 cmH$_2$O CPAP (n=20)	p-value
**Demographical data**			
Gestational age at birth (weeks) [a]	27^{+3} (25^{+5}-28^{+3})	28^{+5} (25^{+4}-29^{+4})	0.588
Birth weight (grams) [a]	1047 (905-1205)	935 (757-1180)	0.328
Gender (% male) [b]	5 (50%)	12 (60%)	0.705
Type of pregnancy (n, % twin) [b]	2 (20%)	10 (50%)	0.235
Mode of delivery (n, % caesarean section) [b]	1 (10%)	10 (50%)	0.049
Antenatal steroids			
Course started (n, %) [b]	10 (100%)	18 (90%)	0.540
Course completed (n, %) [b]	8 (80%)	13 (73%)	0.675
Maternal medication use influencing infants respiration e.g. general anaesthesia (n, %) [b]	0 (0%)	1 (5%)	1.000
Complications during pregnancy (n, %) [b]	4 (40%)	11 (55%)	0.700
Preterm prelabour rupture of membranes (n, %)	3 (30%)	6 (30%)	
Pregnancy-induced hypertension (n, %)	1 (10%)	4 (20%)	
Intra-uterine infection (n, %)	1 (10%)	5 (25%)	
Intra-uterine growth restriction (n, %)	0 (0%)	3 (15%)	

Multiple (n, %)	1 (10%)	7 (35%)	
Apgar score ‘1 [a]	5 (2-7)	6 (3-8)	0.594
Physiological based cord clamping (n, %) [b]	3 (30%)	5 (25%)	1.000
**Primary outcome**			
SpO_2 (%) min 2-5 after birth [c]	62 (52-70)	64 (47-74)	0.922

Data analysed per intention-to-treat. As for the demographical data: numerical data presented as median (Q1-Q3) compared using a Kruskal-Wallis test [a] and categorical data presented as n, (%) compared using a Fisher's exact test [b]. The primary outcome is presented as median (Q1-Q3) compared using a linear regression mixed model [c].
